# Malaria morbidity and immunity among residents of villages with different *Plasmodium falciparum *transmission intensity in North-Eastern Tanzania

**DOI:** 10.1186/1475-2875-3-26

**Published:** 2004-07-28

**Authors:** John PA Lusingu, Lasse S Vestergaard, Bruno P Mmbando, Chris J Drakeley, Caroline Jones, Juma Akida, Zacharia X Savaeli, Andrew Y Kitua, Martha M Lemnge, Thor G Theander

**Affiliations:** 1 National Institute for Medical Research, Amani Medical Research Centre, Amani & NIMR Headquarters, Dar es Salaam, Tanzania; 2 Centre for Medical Parasitology at Institute of Medical Microbiology and Immunology, University of Copenhagen, and Department of Infectious Diseases, Copenhagen University Hospital (Rigshospitalet), Copenhagen, Denmark; 3 Department of Infectious and Tropical Diseases, London School of Hygiene and Tropical Medicine, London, UK

## Abstract

**Background:**

The relationship between the burden of uncomplicated malaria and transmission intensity is unclear and a better understanding of this relationship is important for the implementation of intervention programmes.

**Methods:**

A 6-month longitudinal study monitoring risk factors for anaemia and febrile malaria episodes was conducted among individuals aged below 20 years, residing in three villages of different altitude in areas of high, moderate and low malaria transmission intensity in North-Eastern Tanzania.

**Results:**

The burden of anaemia and malarial fever fell mainly on the youngest children and was highest in the village with high transmission intensity. Although a considerable percentage of individuals in all villages carried intestinal worms, logistic regression models indicated that *Plasmodium falciparum *was the only significant parasitic determinant of anaemia. Interestingly, children who carried low-density parasitaemia at the start of the study had a lower risk of contracting a febrile malaria episode but a higher risk of anaemia during the study period, than children who were slide negative at this point in time.

**Conclusion:**

Young children living in the high transmission village carried a very high anaemia burden, which could be attributed to malaria. The overall incidence of febrile malaria was also highest in the high transmission village particularly among those under five years of age. These data suggest that in rolling back malaria, available resources in prevention programmes should primarily be focussed on young children, particularly those residing in areas of high malaria transmission.

## Background

*Plasmodium falciparum *malaria remains an important public health problem in sub-Saharan Africa. To develop and assess the efficacy of control measures, it is important to obtain a better understanding of how the malaria disease burden is distributed among population groups and how this burden is affected by changes in malaria transmission intensity [1]. In areas of high malaria transmission infants and young children carry a very high disease burden [2], but protective immunity is developed in early childhood. Adults and older children are able to control parasitaemia and therefore only rarely suffer from mild malaria symptoms [3,4]. In areas of low malaria transmission, immunity develops slowly and malaria affect all age groups [5,6]. It has been suggested that the societal burden of malaria does not necessarily increase with transmission intensity, but peaks at a certain level of transmission after which it remains constant and may even decrease [7-9]. To address this issue we have compared the malaria situation in three communities situated North-Eastern Tanzania, which show differences in transmission intensity. In this area, transmission intensity is determined by altitude and large differences in transmission can be found within a limited geographical area [10-12]. This study reports the results of six months morbidity follow-up, during which the incidence of febrile malaria episodes and the prevalence of anaemia were assessed in cohorts of 0–19 year old individuals.

## Methods

### Study area

The study was conducted in three villages in Tanga region, North-Eastern Tanzania. The three villages were Mgome (5°12'S, 38'51'E) at an altitude of approximately 200 meters, Ubiri (4°72'S, 38°29'E) at an altitude of approximately 1,200 meters, and Magamba (4°75'S, 38°29'E) at an altitude of approximately 1,700 meters.

The climate in the area is characterized by variations in rainfall and temperature related both to season and altitude [12]. The long rainy period occurs during April-May, while short rains occur in November-December. Mean daily temperatures are highest in January and lowest in July. Generally, the malaria transmission season peaks just after the rainy seasons with most consistent transmission in lowland sites from April to July. Previous studies have reported parasite prevalence rates to be in the ranges of 79–90% in the lowlands, 27–46% at intermediate altitudes and 8–16% in the highlands [10]. Entomological surveys in the study areas have shown that *Anopheles gambiae *is the most prevalent vector in the lowlands, while *Anopheles funestus *predominates in the highlands [10]. The entomological inoculation rates (EIR) have been reported to be in the range between 91–405 in the lowlands, and between 1.8–34 at intermediate altitudes [10]. In the highlands, mosquito densities are too low to allow reliable EIR measurements, but an EIR of 0.03 has been extrapolated [10]. Villagers living at low and intermediate altitudes perceive malaria as a major problem among both children and adults, but at the highest altitudes villagers consider that malaria is not a major part of the disease burden in either adults or children. There is little difference in treatment seeking behaviour for febrile illness between the altitudes. Treatment is generally sought for symptoms rather than for the disease and first treatment is almost universally an anti-pyretic drug bought from local shops (Caroline Jones, unpublished data). For all three villages, the nearest health facility is located within a distance of 13 km. Mgome is served by Umba Dispensary (10 km), Masaika Dispensary (5 km), Mkuzi Health Centre (7 km) and Muheza Designated District Hospital (14 km). Ubiri village is served by Lushoto District Hospital at a distance of approximately 13 km. Magamba village has a government and a private missionary dispensary both within the village, and is served also by Lushoto District Hospital at a distance of about 15 km. At the time of the study, sulphadoxine-pyrimethamine (SP) was the first-line treatment for uncomplicated malaria in Tanzania. It has been documented that the level of SP resistance is high in the Mgome area [13], whereas the situation has not been monitored previously in Ubiri and Magamba.

Land use in the lowland areas is characterized by subsistence farming of maize, rice, bananas, beans, cassava, coconuts, fruits and other crops, as well as large-scale production of sisal. In the highlands, there is subsistence farming, mainly of maize, beans, bananas, potatoes, cabbages, tomatoes and fruits, and also large-scale production of tea and coffee.

### Study population

Prior to the study, census surveys were done in each village and study individuals randomly selected from a census list. Mgome village is inhabited mainly by the Bondei tribe (60%), while Ubiri and Magamba are inhabited by Sambaa at 97% and 57%, respectively. The aim was to recruit a total of 250 individuals below the age of twenty years from each village, distributed in different age groups as follows: 0–1 year: n = 25, 1 year: n = 25, 2 years: n = 25 3 years: n = 25, 4 years: n = 25, 5–6 years: n = 25, 7–9 years: n = 25, 10–14 years: n = 40 and 15–19 years: n = 40.

### Cross-sectional surveys

Malariometric surveys were conducted in each village in April, July and September 2001. During the first survey, the purpose of the study was explained and consent to participate obtained from each study individual or their parents/guardians. Baseline demographic data were collected together with a history of migration and recent movements. The use of malaria preventive measures was also recorded. A history of recent illness was obtained, emphasizing symptoms suggestive of malaria. Physical examination on signs related to malaria such as temperature, pulse, spleen size, pallor and respiratory rate was conducted. Axillary temperature was measured using digital thermometers. Height, weight and upper-arm-circumference were recorded for estimation of nutritional status. For any individual diagnosed with mild disease, appropriate drugs were administered in the field. Individuals with symptoms of malaria were treated with SP. Participants with severe disease were referred to the nearby hospital.

Five millilitres of venous blood were collected from study individuals aged three years and above into vacutainer tubes containing citrate buffer. For children below three years, 300–400 μl of capillary blood from a fingerprick were collected into eppendorf tubes containing EDTA. The haemoglobin (Hb) of each participant was measured from drops of blood using a HemoCue^® ^photometer (Ångelholm, Sweden). Whole blood was used to prepare thick and thin blood smears for malarial microscopy. These were stained with 10% Giemsa stain for 15–20 minutes after fixing thin smears with methanol. Asexual and sexual parasites were counted against 200 and 500 white blood cells, respectively. The differentiation of malaria parasite species was confirmed by microscopy of thin smears. A blood smear was declared negative only after examination of 200 high power fields. The density of asexual parasites was calculated assuming 8000 leucocytes per μl of blood and expressed as parasites per μl.

During the first cross-sectional survey, study participants were asked to collect stool and urine specimens in special containers. Direct smear-technique was used to check for the presence of hookworm ova and other intestinal parasites. A pinhead of stool was collected, put on a slide and emulsified in a drop of normal saline. A cover slip was then applied and the slide examined using low-power microscopy.

### Longitudinal monitoring of febrile episodes

Local village helpers (two community members per village) and health workers at nearby health facilities performed passive case detection during the 6-month study period. The village helpers were provided with first-line antimalarial drugs (SP), paracetamol, slides, blood lancets, treatment charts, febrile case detection forms and storage boxes. Villagers could seek treatment at any time from these helpers. Patients with symptoms of malaria were treated with first-line antimalarial drugs or, if they had severe symptoms or did not respond adequately to the first-line treatment, they were referred to a health facility. Prior to treatment the village helpers collected clinical information and a malaria blood smear.

At each nearby health facility, two permanent staff members monitored study participants seeking medical treatment at the facility. If a study participant presented at the facility with history of fever and/or an axillary temperature ≥ 37.5°C, a form was completed and a blood smear collected. Once per month active febrile case detection was undertaken by the research team. During active case detection, each study participant was seen by a trained physician and a blood smear was taken from any study participant reporting a history of fever within two days and/or those who had an axillary temperature ≥ 37.5°C

### Case definitions

Anaemia was defined as haemoglobin < 11.0 g/dl [14,15]. To adjust for the physiological effect of altitude on haemoglobin concentration, a correction factor was calculted with haemoglobin values being normalized to sea level for direct comparison between the study villages. The correction factor assumed a linear relationship between increasing altitude and haemoglobin, although the relationship may not necessarily always be exact [16]. For Mgome (200 m), the correcting factor was a reduction of 0.1 g/dl, for Ubiri (1,200 m) the factor was 0.8 g/dl and for Magamba (1,700 m) the factor was a reduction of 1.0 g/dl. Febrile malaria episodes were defined as an axillary temperature ≥ 37.5°C and /or a history of fever within the previous 48 hours in the presence of asexual *P. falciparum *parasites above a defined density cut-off level. Many individuals carried low density asymptomatic parasitaemia, and fever among parasitaemic individuals may also have been caused by other illness [17]. Thus, to account for the variation in levels and point prevalence of asymptomatic parasitaemia between study villages [18–20], as well as the different age groups involved in the study [21], different *P. falciparum *density cut off levels were applied in each village. To balance between sensitivity and specificity in diagnosing a febrile malaria episode, we aimed at a febrile malaria case specificity >80%. In Magamba (the low transmission village), a cut-off of 40 parasites/μl was applied, while cut-offs of 1000 parasites/μl and 5000 parasites/μl were used in Ubiri (the moderate transmission village), and Mgome (the high transmission village), respectively. Age-specific incidence rates of febrile malaria episodes were calculated as the number of episodes divided by the number of days that individuals in the age group were at risk during the follow-up. After a febrile malaria episode an individual was censored for 28 days [6]. The effect of using different parasite density cut-offs in the definition of a febrile episode was evaluated by not applying a cut-off in the definition or by applying age specific cut-offs [21].

### Statistical methods

All data were double-entered into a database in Epi-lnfo Version 6.04d (CDC, Atlanta, USA) and statistical analyses were performed with Stata version 8 (Stata Corporation, Texas, USA). Univariate analyses and multivariate logistic regression were performed to determine risk factors for anaemia and febrile malaria episodes.

For Mgome village, a logistic regression model was developed to determine whether the result of the first slide reading in April could be used to predict the subsequent risk of developing anaemia or febrile malaria during the following six months of morbidity surveillance. In this model, *P. falciparum *parasitaemia was categorised as no parasitaemia if no parasites were detected microscopically, low-density if parasitaemia was between 40 parasites/μl and 4999 parasites/μl, and high-density if the level was above or equal to 5000 parasites/μl. Thus, the first slide reading of individuals who did not have fever/had normal haemoglobin levels at enrolment was used to predict the risk of developing a subsequent episode of malarial fever/anaemia.

### Ethical considerations

Ethical clearance was granted by the Medical Research Co-ordinating Committee of the National Institute for Medical Research, Tanzania. Prior to the study, meetings were held with local authorities and with the villagers in each study village, during which the aims of the study were explained. Informed consent documents for the study were prepared in English and translated into Kiswahili before administration to both village leaders and participants. Written informed consent to participate was obtained from each study individual or from his/her parents or guardians. Study individuals were free to withdraw from the study at any time without giving any reasons, or being disqualified from any medical services that were provided to all villagers throughout the study period. At the end of the study, preliminary findings were presented at village meetings.

## Results

### Prevalence and densities of Plasmodium species and other infections

Three study villages were selected to represent areas of markedly different malaria transmission intensity. In each village, approximately 250 individuals under the age of 20 years were recruited. Few individuals reported using anti-malarial preventive measures (Table 1). Repeat investigations on the same individuals were undertaken at enrolment in April 2001, and during subsequent cross-sectional surveys in July and September 2001. Only about 10% of the study participants were lost to follow-up in each village.

**Table 1:** Baseline characteristics of the study villages

**Baseline characteristic**	**Mgome**	**Ubiri**	**Magamba**
Altitude (m) [range]	196 [165, 208]	1216 [1174, 1262]	1585 [1659, 1751]
Enrolled (0–19 years) (Male/Female)	254 (115/139)	250 (139/111)	255 (132/123)
Use of preventive measures			
Nets (%)	18/254 (7.1)	5/250 (2.0)	14/255 (5.5)
Burning coils (%)	8/254 (3.1)	1/250 (0.4)	0/255 (0)
Neem (%)	0/254 (0)	1/250 (0.4)	0/255 (0)
Spray (%)	0/254 (0)	0/250 (0)	3/255(1.2)
Prophylaxis (%)	0/254 (0)	1/250 (0.4)	1/255 (0.4)

**Figure 1:**
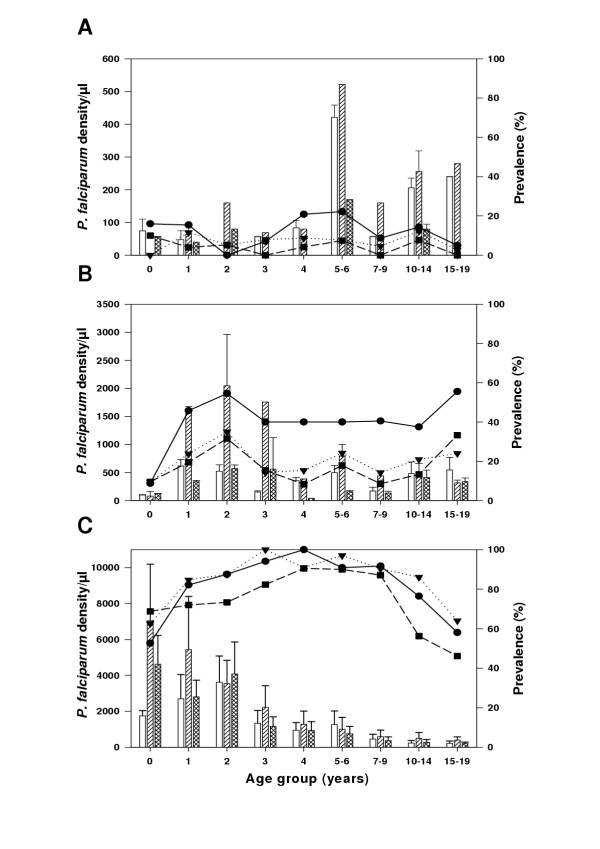
**Age-specific *P. falciparum *prevalence and geometric mean densities (positives only) by village and season. **Panels A, B, and C show age-specific *P. falciparum *densities and prevalence in Magamba (low transmission), Ubiri (moderate transmission) and Mgome (high transmission), respectively. Lines indicate the prevalence rate for each survey. Solid lines with filled circle for April 2001, dotted lines with filled triangle for July 2001, and dashed lines with filled box for September 2001. Bars indicate *P. falciparum *densities (positives only) for each survey. Empty bars indicate the April 2001 surveys, hatched bars indicate the July 2001 surveys, and crossed hatched bars indicate the September 2001 surveys. Error bars indicate 95% confidence interval.

As expected, *P. falciparum *prevalence and parasite densities (Figure 1) were higher in Mgome than in Ubiri and Magamba (trend test, z = 15.64, p < 0.001). In Mgome, the carrier rate was particularly high for children aged 1–9 years and then declined in the older age groups (trend test, z = -3.2, p < 0.001). In Ubiri, carrier rates were low in infants, peaked at the age of two years, but showed little variation in the age groups between 4 and 19 (Figure 1). Although carrier rates in Ubiri were slightly higher in April than in July and September, there were no marked seasonal changes in carrier rate by age in any of the villages. The parasite densities in those carrying parasites did not differ between villages after the age of six years. Among the under fives, children from Mgome and Ubiri carried higher levels of parasitaemia in July than in April and September 2001 surveys. Interestingly, between April and July surveys, there was a marked difference in the age-specific pattern of parasite density in Mgome. In April, the peak parasite density was noted in age group of 2 years, whereas the youngest had the highest parasite density in July surveys. Based on these findings, we categorised Mgome as a high transmission (holoendemic) village, Ubiri as a moderate transmission (mesoendemic) village, and Magamba as low transmission (hypoendemic) village. *P. falciparum *was the most predominant malarial parasite accounting for more than 95% of all malaria infections. The other malaria species were mainly found as mixed infections. The April prevalence rates of *Plasmodium malariae *in Mgome and Ubiri were 8.3 % and 3.9%, respectively, while these rates for *Plasmodium ovale *was 1.0% and 0%. In Magamba, only *P. falciparum *was found.

A total of 492 individuals from the three villages submitted stool and urine samples, which were investigated for worms. Worms were found in 35.0% of study participants living in Mgome, and in 29.2% and 7.8% of individuals from Ubiri and Magamba, respectively.

In Mgome, spleen enlargement was common (about 49.21%) and associated with age (Spearman rho = 0.238, p < 0.001) while in the two other villages the prevalence of splenomegaly was low and with no distinct age-pattern (data not shown). This distribution of splenomegaly remained stable during the study period.

### Haemoglobin levels and anaemia

**Figure 2:**
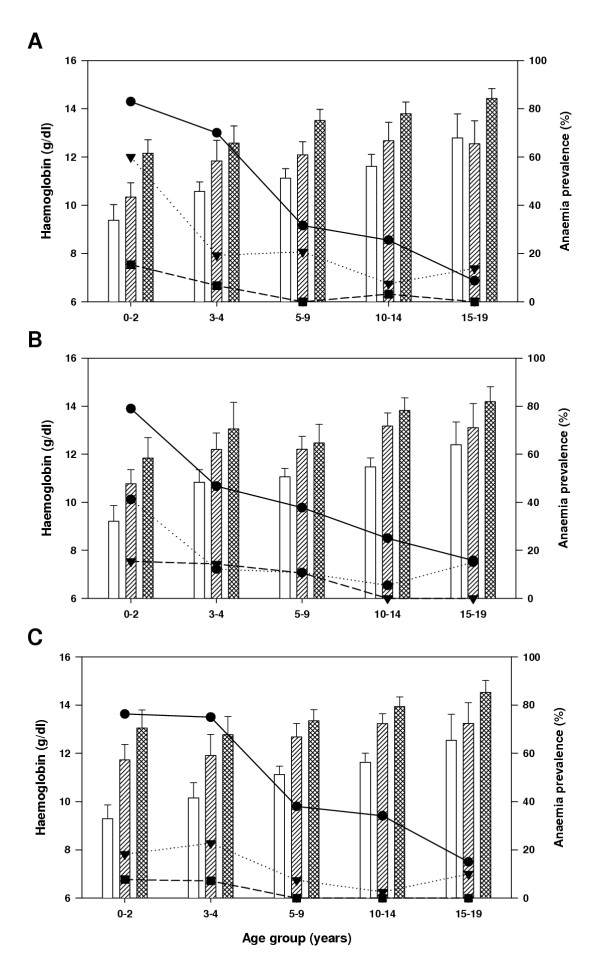
**Age-specific anaemia prevalence and mean haemoglobin levels by village and season. **Panels A, B, and C show results of surveys conducted in April, July and September 2001, respectively. The lines and symbols show patterns of anaemia prevalence for each village (Mgome: solid lines with filled circle, Ubiri: dotted lines with filled triangle, Magamba: dashed lines with filled square). Bars indicate mean altitude adjusted haemoglobin levels (g/dl) and 95% confidence intervals in each village (Mgome: empty bars, Ubiri: hatched bars, Magamba: crossed hatched bars).

Haemoglobin levels were measured in all 759 individuals during enrolment and among those who reported for the subsequent cross-sectional surveys in July and September 2001. Regardless of the season, haemoglobin levels increased with both altitude and age (Figure 2). Univariate analysis indicated that age, altitude of residence, and presence of *P. falciparum *parasitaemia were associated with anaemia (Table 2) and this was supported by multivariate analyses in which *P. falciparum *was the only parasitic infection showing a statistically significant association to anaemia (Table 2).

**Table 2:** Crude and adjusted odds ratios for risk factors for anaemia

**Explanatory variable**	**Crude odds ratio (95% Cl)**	**p-value**	**Adjusted odds ratio (95% Cl)**	**p-value**
Age group (years)				
0–2	21.38 (8.36–54.65)	<0.001	20.41 (7.44 – 56.0)	<0.001
3–4	7.38 (2.82–19.25)	<0.001	5.42 (1.93–15.20)	<0.001
5–9	3.43 (1.36–8.66)	0.009	2.18 (0.82–5.81)	0.118
10–14	1.86 (0.70–4.99)	0.215	1.51 (0.54–4.27)	0.435
15–19	1		1	
Sex				
Male	1.04 (0.70 – 1.54)	0.85	1.16 (0.71 – 1.90)	0.551
Female	1		1	
Village				
Mgome	19.99 (7.08–56.42)	<0.001	15.55 (4.78–50.65)	<0.001
Ubiri	8.02 (2.80–23.82)	<0.001	6.44 (2.08–19.92)	0.001
Magamba	1		1	
Parasites				
*P. falciparum*	3.77 (2.44 – 5.83)	<0.001	2.0 (1.11–3.62)	0.021
Hookworm	1.41 (0.83–2.39)	0.201	1.43 (0.76–2.69)	0.264
Ascariasis	0.86 (0.52–1.44)	0.564	1.04 (0.54–1.92)	0.952
Amoeba	0.16 (0.02–1.20)	0.074	0.16 (0.02–1347)	0.091
Schistosoma	0.42 (0.12–1.46)	0.174	0.28 (0.07–1.20)	0.086

### Malaria morbidity during follow-up

**Figure 3:**
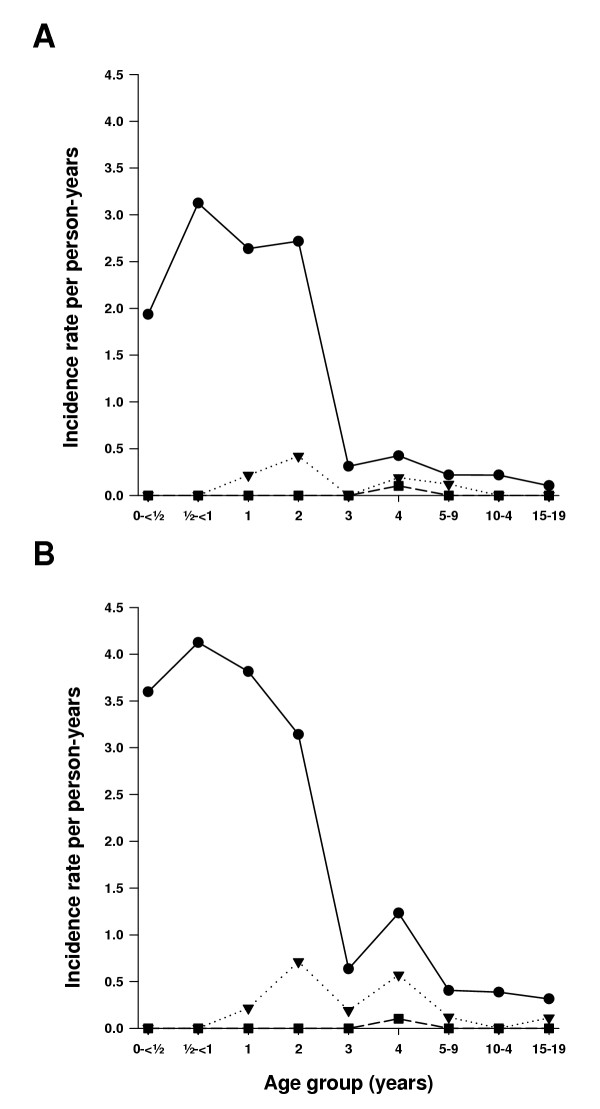
**Incidence rates of febrile malaria episodes by age group. **Panel A: Incidence rates calculated using village specific parasite density cut-offs in the definition of episodes. In Mgome (solid lines with filled circle) the cut-off was 5000 parasites per μl, in Ubiri (dotted lines with filled triangle) 1000 parasites per μl, and in Magamba (dashed lines with filled square) 40 parasites per μl. Panel B: Incidence rates calculated using a definition in which all fevers accompanied by positive slide were considered a febrile malaria episode.

Of the 759 individuals enrolled in the study, 669 (88%) adhered to the follow-up scheme and were included in the analysis of febrile episodes. Loss to follow-up was due to death (three individuals) or emigration. Using the village-specific density cut-off described above, 54 individuals had febrile malaria episodes in Mgome, 10 in Ubiri and none in Magamba during the six-month follow-up period. The mean age of febrile malaria individuals was 1.97 years (95% Cl: 1.50, 2.59) and 3.23 years (95% Cl: 1.59, 5.86) for Mgome and Ubiri, respectively. Children below five years carried the major burden of febrile malaria episodes in Mgome (Figure 3, panel A). The data was also analysed using age-specific parasite cutoffs [21] in the case definition (data not shown) and using a definition in which all fevers accompanied by a positive slide were considered a febrile malaria episode (Figure 3, panel B). The latter definition increased the incidence rates in Mgome and Ubiri slightly, but the overall conclusion that the incidence rates were by far the highest in the children under five years living in Mgome was not affected by the case definition used.

In Mgome, host age and the presence of low-density parasitaemia at the start of the study were consistently found to be associated with decreased risk of suffering a febrile malaria episode during the morbidity follow-up. Other variables such as sex, splenomegaly and use of a mosquito net did not contribute significantly to the model. In logistic regression models correcting for age, those who carried parasites at low densities in April had a four-fold lower risk (P < 0.03) of developing a febrile malaria episode during follow-up than those who were slide negative (Table 3).

**Table 3:** Logistic regression model showing the risk of developing a febrile malaria episode during the 6 month morbidity surveillance according to age and the result of the slide at the initiation of the study in Mgome

**Explanatory variable**	**Crude odds ratio (95% Cl)**	**p-value**	**Adjusted odds ratio (95% Cl)**	**p-value**
Low parasite density^1^	0.34 (0.15–0.78)	0.011	0.22 (0.06–0.89)	0.033
High parasite density^2^	3.0 (1.09–8.29)	0.034	1.36 (0.27–8.82)	0.706
No parasitaemia^3^	1		1	
Age (years)	0.66 (0.58 – 0.76)	<0.001	0.51 (0.332 – 0.772)	0.002
Age squared	0.98 (0.97 – 0.998)	0.007	1.03 (1.007–1.047)	0.007

^1^Parasitaemia in April between 40 and 4999 parasites/μl ^2^Parasitaemia in April >4999 parasites/μl ^3^Slide negative in April

In Mgome, 112 of the 254 individuals were not anaemic on enrolment in April 2001. Out of these, 68 (mean age (years) and 95% Cl: 11.2 [10.2, 12.3]) had normal haemoglobin levels during the July and September cross sectional surveys, while 44 developed anaemia during the study (mean age (years) and 95% Cl: 7.9 [6.4, 9.5]). Logistic regression models correcting for age showed that the risk of developing anaemia during the study was 4.4 times (p = 0.038) higher in individuals carrying low-density parasitaemia in April than in those who were slide negative (Table 4).

**Table 4:** Logistic regression model showing the risk of developing anaemia during the 6 month morbidity surveillance according to age and the result of the slide at the initiation of the study in Mgome

**Explanatory variable**^1^	**Crude odds ratio (95% Cl)**	**p-value**	**Adjusted odds ratio (95% Cl)**	**p-value**
Low parasite density	3.98 (1.276–13.095)	0.02	4.38 (1.10–17.69)	0.038
High parasite density	3.17 (0.601–16.692)	0.17	2.43 (0.35–16.73)	0.369
No parasitaemia	1		1	
Age (years)	0.86 (0.789 – 0.94)	0.001	0.51 (0.332–0.772)	0.002
Age squared	0.99 (0.99 – 0.998)	0.007	1.03 (1.007–1.047)	0.007

^1^Refer to table 3

## Discussion

This prospective longitudinal study was designed to compare the burden of uncomplicated malaria in three similar villages situated in areas of markedly different transmission intensity. Not surprisingly, the study showed that individuals living in the village with very high malaria transmission carried a markedly higher burden of both anaemia and febrile malaria episodes compared to villagers at the sites with lower transmission. This result is in agreement with results from previous studies in the area [11]. The villages in the highlands are prone to malaria epidemics [12], but such epidemics did not occur during the study period. If they had, it is conceivable that the incidence of febrile malaria episodes at these sites would have reached or even exceeded the incidence found in the high transmission village [6,9]. Never the less, the anaemia burden in the high transmission village was very high among infants and young children. The burden among these children was much greater than among individuals of the corresponding age groups in the other two villages. During the first survey, villagers were also investigated for the presence of parasites in urine and faeces, but neither of these was shown to be a significant risk factor for anaemia in the multivariate logistic regression models (Table 2). Thus, the difference in anaemia burden between the sites appears likely to have been due to differences in malaria transmission intensity. The reason that hookworm infection did not constitute a risk factor for anaemia is likely to be a consequence of the fact that the villagers receive regular deworming medication as part of health promotion programmes, and therefore, the worm burden was rather low [22]. The heavy burden of anaemia carried in populations exposed to high malaria transmission has recently been highlighted [14]. The results of this study support the findings that malaria plays a major role in the burden of anaemia and these results are further corroborated by the fact that malaria interventions such as insecticide treated nets and intermittent preventive treatment in infants (IPTi) considerably reduce the incidence of anaemia [23-25]. From a public health perspective, our results reinforce the view that malaria prevention programmes should focus their attention on high-transmission areas and concentrate particularly on children under five years of age. Our study was not designed to compare incidences of severe disease and malaria deaths. It has previously been suggested that this malaria burden is in fact higher in populations exposed to moderate transmission than in populations living in areas of very high transmission [8]. The results of a large hospital-based study recently conducted in North-Eastern Tanzania over a wide range of transmission intensities suggested, however, that there was a positive correlation between severe malaria outcomes and intensity of transmission (Reyburn et al., submitted for publication).

The longitudinal design of our study allowed an exploration of whether *P. falciparum *carriage at the beginning of the study influenced the risk of developing febrile malaria episodes or anaemia during the following study period. Interestingly, multivariate logistic regression models indicated that children carrying low-density parasitaemia during the first cross sectional survey were at a lower risk of developing a febrile malaria episode than children without detectable parasitaemia or children with higher levels of parasitaemia. This apparent protective effect of low-grade parasitaemia was recently also reported in a study from Ghana [26], but in our study this protection came with a price since children who controlled the parasite density at low levels were at markedly higher risk of developing anaemia.

## Conclusions

The overall burden of malaria morbidity was found to be highest in the high-transmission area, where infants and children carried a very high malaria burden in the form of febrile episodes and anaemia. Populations in the areas of moderate and low transmission suffered a significantly lower morbidity. Therefore, in order to roll back malaria, available resources in malaria control programmes should focus on underfives residing in the high-transmission areas.

## Authors' contributions

JPAL and LSV participated in the planning of the study, carried out field surveys, analysed the data and drafted the manuscript. BPM participated in designing the study, carried out field surveys and managed the data. CJD participated in study planning, in the fieldwork and in editing of the manuscript. CJ participated in the planning of the study and conducted the socio-economic analysis of study villages. JA and ZXS participated in the field surveys and performed microscopy of all blood smears. AYK, MML and TGT participated in study planning, coordination, and analysis of data. All authors participated in the paper writing and approved the final manuscript.
